# SUMOylation of the nuclear pore complex basket is involved in sensing cellular stresses

**DOI:** 10.1242/jcs.224279

**Published:** 2019-04-03

**Authors:** Hanne Folz, Carlos A. Niño, Surayya Taranum, Stefanie Caesar, Lorenz Latta, François Waharte, Jean Salamero, Gabriel Schlenstedt, Catherine Dargemont

**Affiliations:** 1 Institute of Medical Biochemistry and Molecular Biology, Universität des Saarlandes, D-66421 Homburg, Germany; 2 Université Paris Diderot, Sorbonne Paris Cité, Pathologie et Virologie Moléculaire, INSERM, CNRS, Hôpital St. Louis, 75475 Paris, France; 3 Institut Curie, PSL Research University, CNRS UMR 144, UPMC, Space-time Imaging of Organelles and Endomembranes Dynamics & PICT-IBiSA Imaging Core Facility, 75005 Paris, France

**Keywords:** Nuclear pore complex, SUMO, Nuclear basket, Osmotic stress, Genotoxic stress

## Abstract

The nuclear pore complex (NPC) is the major conduit for nucleocytoplasmic transport and serves as a platform for gene regulation and DNA repair. Several nucleoporins undergo ubiquitylation and SUMOylation, and these modifications play an important role in nuclear pore dynamics and plasticity. Here, we perform a detailed analysis of these post-translational modifications of yeast nuclear basket proteins under normal growth conditions as well as upon cellular stresses, with a focus on SUMOylation. We find that the balance between the dynamics of SUMOylation and deSUMOylation of Nup60 and Nup2 at the NPC differs substantially, particularly in G1 and S phase. While Nup60 is the unique target of genotoxic stress within the nuclear basket that probably belongs to the SUMO-mediated DNA damage response pathway, both Nup2 and Nup60 show a dramatic increase in SUMOylation upon osmotic stress, with Nup2 SUMOylation being enhanced in Nup60 SUMO-deficient mutant yeast strains. Taken together, our data reveal that there are several levels of crosstalk between nucleoporins, and that the post-translational modifications of the NPC serve in sensing cellular stress signals.

## Abstract (precis)

**Summary:** Post-translational modifications, and in particular SUMOylation, of the nuclear basket subcomplex of the nuclear pore complex serve in its function as a sensor for mediating cellular stress signals.

## INTRODUCTION

One of the defining features of a eukaryotic cell is the presence of membrane-enclosed organelles to carry out specialized functions. The nucleus is the largest cell organelle, housing its DNA, and is separated from the cytoplasm by a double membrane called the nuclear envelope (NE). The nuclear pore complexes (NPCs), specialized substructures present in the nuclear envelope, serve as the gatekeepers for selective RNA and protein transport between the nucleus and the cytoplasm (Beck and Hurt, 2017).

First observed in the 1950s as small dense regions dotting the NE, the NPCs are now known to be massive megadalton-sized multi-protein assemblies that are embedded at points where the outer and inner nuclear membranes of the NE fuse. They comprise ∼30 nucleoporin proteins (Nups) that are arranged in multiple copies within defined subcomplexes, and there is evidence that the stability of these subcomplexes is higher than the supramolecular complex (Cronshaw et al., 2002; Rout et al., 2000; Schwartz, 2016). The NPCs are highly conserved across species, though the number of NPCs per cell varies widely in different species. For example, nuclei of HeLa cells contain ∼3000 NPCs while yeast nuclei contain ∼100–200 NPCs.

Electron microscopy studies show that NPCs exhibit an eightfold rotational symmetry with cytoplasmic, inner and nucleoplasmic rings. The Y complex is another essential component of the NPC, and serves as a scaffold in its assembly. Indeed, depletion of this complex abolishes NPC formation. The NPC is tethered to the NE by a set of transmembrane Nups that are poorly conserved across species. In addition, there are eight rod-shaped extensions from the NPC that connect to a distal ring on the nucleoplasmic side, and form the nuclear basket (Hoelz et al., 2016; Knockenhauer and Schwartz, 2016). Recent breakthrough studies on NPC structure have delineated the architecture of these behemoth protein complexes at sub-nanometer precision in yeast cells. These investigations show that the NPC comprises a ring structure with rigid diagonal columns and flexible connectors that confer strength and resilience, and bring the discrete sub-regions together (Kim et al., 2018).

The nuclear basket in the budding yeast *Saccharomyces cerevisiae* is an assembly of five nucleoporins, namely Nup60, Nup1 and Nup2 of the FG (phenylalanine/glycine-rich repeats containing) nucleoporin subfamily, and two myosin-like proteins Mlp1 and Mlp2. In vertebrates, Nup153, Nup50 and Tpr together form the nuclear basket assembly (Dilworth et al., 2001; Strambio-de-Castillia et al., 1999). Interestingly, both Nup60 and Nup1 present N-terminal amphipathic helices that mediate tethering to the nuclear envelope (Mészáros et al., 2015). In addition, we have previously shown that modification of Nup60 by ubiquitin facilitates its interaction with the Nup84 component of the Y complex and thus participates in the tethering of the nuclear basket to the core NPC (Niño et al., 2016). Nup60 (and its vertebrate homolog Nup153) are important for the localization of Nup2 (and its vertebrate homolog Nup50) at the NPC, interaction with the SUMO protease Ulp1, and recruitment of Mlp1 and Mlp2 at the NPC (Denning et al., 2001; Dilworth et al., 2001; Zhao et al., 2004). Functionally, Nup60, Nup1 and Nup2 facilitate docking of transport complexes to the NPC. In addition, Nup2 (and Nup50 in vertebrates), are required for classical NLS protein import and importin α recycling (Guan et al., 2000; Matsuura and Stewart, 2005; Solsbacher et al., 2000).

Initial studies on the functions of NPCs focused on their role as conduits in nucleocytoplasmic transport. In recent years, several studies have shed light on their transport-independent functions, including gene regulation, chromatin organization, DNA repair, RNA processing, RNA quality control and cell cycle regulation (reviewed in Raices and D'Angelo, 2017 and Simon et al., 2018). Interestingly, several nucleoporins are known to undergo post-translational modifications (PTMs), including ubiquitylation, SUMOylation and phosphorylation, that could account for this functional plasticity of the NPC. Our published studies indicate that over 50% of Nups are ubiquitylated, most by monoubiquitylation, suggesting a non-degradative role of this PTM in the regulation of NPC structure and function (Hayakawa et al., 2012; Niño et al., 2012). We have previously shown that the dynamic nature of PTMs, specifically ubiquitylation of the yeast nuclear basket protein Nup60, regulates the plasticity of the NPC and contributes to its function in nuclear metabolism. Preventing the ubiquitylation of Nup60 affects the association of Nup60 and its partner Nup2 with Nup84, and renders the cell vulnerable to genotoxic stress (Niño et al., 2016).

In addition to ubiquitylation, several studies have reported physical interactions between nucleoporins and enzymes of the small ubiquitin-related modifier (SUMO) pathway (Palancade and Doye, 2008). SUMOylation resembles the ubiquitylation process and engages the action of the E1-E2-E3 enzymes [E1 SUMO-activating enzyme complex Aos1–Uba2; E2 SUMO-conjugating enzyme Ubc9 and E3 SUMO-ligases Siz1, Siz2 (also known as Nfi1), Mms21 and Cst9], while deSUMOylation is carried out by the SUMO proteases Ulp1 and Ulp2, with Ulp1 being tethered to the NPC by Nup60 (Zhao et al., 2004). We previously found that Nup60 is SUMOylated by the concerted action of E1 and E2 enzymes, together with Siz1 and Siz2, on two distinct lysine residues, and that SUMOylated Nup60 is clear target of Ulp1 (Niño et al., 2016). SUMOylation and SUMO modifications influence the conformation, stability, localization or function of the target protein (Flotho and Melchior, 2013). Unlike ubiquitylation, however, SUMOylation does not trigger protein degradation. In fact, SUMO is an important player in several cellular processes that encompass signal transduction to DNA damage pathways including base excision repair (BER), nucleotide excision repair (NER) and double strand break (DSB) repair (Zilio et al., 2017).

Here, we focus our studies on the impact of PTMs on the nuclear basket, and in particular Nup60 and Nup2, upon cellular stresses including osmotic stress, with specific attention on SUMOylation. Our findings support the hypothesis that regulation of NPCs plasticity via PTMs serves to modulate its function as a platform for various nuclear functions, and that the NPC acts as a stress sensor serving to transmit extracellular stress signals into the nucleus.

## RESULTS

### The role of Nup60 SUMOylation in the cellular response to DNA damage

**Fig. 1.**
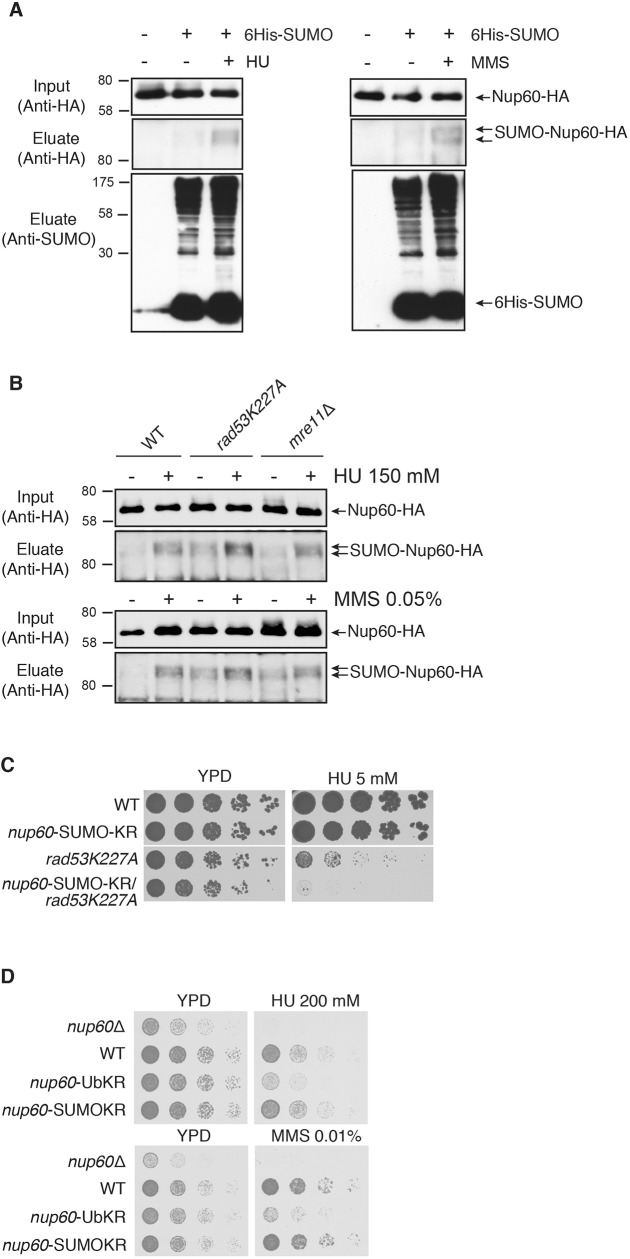
**Nup60 SUMOylation in the DNA-damage response.** (A,B) Ni-purified 6His–SUMO-conjugated forms of Nup60–HA (SUMO–Nup60–HA) were extracted from cells transformed (+) or not transformed (−) with a plasmid encoding 6His–SUMO under control of the *CUP1* promoter and treated (+) or not (−) with HU (150 mM) or MMS (0.05%). Cell lysates (input) and Ni-purified material (eluate) were examined using western blotting with an anti-HA antibody. SUMO expression and efficiency of purification was controlled using an anti-SUMO antibody (bottom) in (A) wild-type cells (*n*=3) and (B) wild-type or mutant strains as indicated (*n*=2). (C) Serial dilutions of wild-type (WT) and indicated mutant cells were spotted on YPD plates without or with HU (5 mM) and grown at 30°C (*n*=4). (D) Serial dilutions of wild-type and indicated mutant cells were spotted on YPD plates without or with HU or MMS at the indicated concentrations and grown at 30°C (*n*=4).

We have previously shown that ubiquitylated Nup60 is a target for Rad53 kinase and reinforces the DNA damage response upon replication stress (Niño et al., 2016). Besides the Mec1- and Rad53-mediated DNA damage response, many DNA repair factors were shown to be SUMOylated in response to DNA damage. Both complementary pathways – phosphorylation and SUMOylation – are necessary to support DNA repair and cell growth upon genotoxic stress (Cremona et al., 2012; Psakhye and Jentsch, 2012). Since Nup60 is not only ubiquitylated but also SUMOylated (Niño et al., 2016), we analyzed whether SUMOylation of genomically HA-tagged Nup60 was affected by such stresses using expression of a copper-inducible 6His-tagged version of yeast SUMO (Smt3), followed by purification from denatured cell extracts on nickel column, and western blot analysis using anti-HA antibodies as previously described (Hayakawa et al., 2012; Niño et al., 2016). Nup60 SUMOylation was induced after exposure to either the DNA synthesis inhibitor hydroxyurea (HU) or the DNA-damaging agent methyl methane sulfonate (MMS) (Fig. 1A). The DNA damage sensor and repair complex MRX has been proposed to favor DNA stress-induced SUMOylation for a subset of repair targets (Cremona et al., 2012). As shown in Fig. 1B, DNA damage-induced Nup60 SUMOylation was independent of Rad53 and the MRX complex subunit Mre11. We previously determined that Nup60 contains two SUMOylation sites within its C-terminus on Lys440-442 and Lys505, and developed a corresponding SUMO-deficient mutant of Nup60 (*nup60*-SUMO-KR; Niño et al., 2016). Interestingly, *nup60*-SUMO-KR and the kinase-dead *rad53K227R* mutations showed additive growth defects upon genotoxic stress when combined in the same strain, indicating that Nup60 belongs to the SUMO-mediated DNA damage response pathway that is independent of the canonical DNA damage response (Fig. 1C). Nevertheless, Nup60 SUMOylation, unlike Nup60 ubiquitylation, had no significant effect on recombination of eroded telomeres (not shown), and, in contrast to Nup60 ubiquitylation (*nup60*-UbKR) (Niño et al., 2016), preventing Nup60 SUMOylation did not cause cell growth defects upon HU or MMS treatment unless combined with mutation of Rad53 (Fig. 1C,D). This weak phenotype is shared by many repair factors. DNA damage indeed triggers a SUMOylation wave promoting modifications of many repair proteins. Inhibiting modification of an individual repair factor is not sufficient to induce a significant phenotype, whereas simultaneous mutations of the SUMO sites of several proteins of the same pathway can lead to major DNA repair defects (Psakhye and Jentsch, 2012).

### Ubiquitylation and SUMOylation of the nuclear basket proteins

**Fig. 2.**
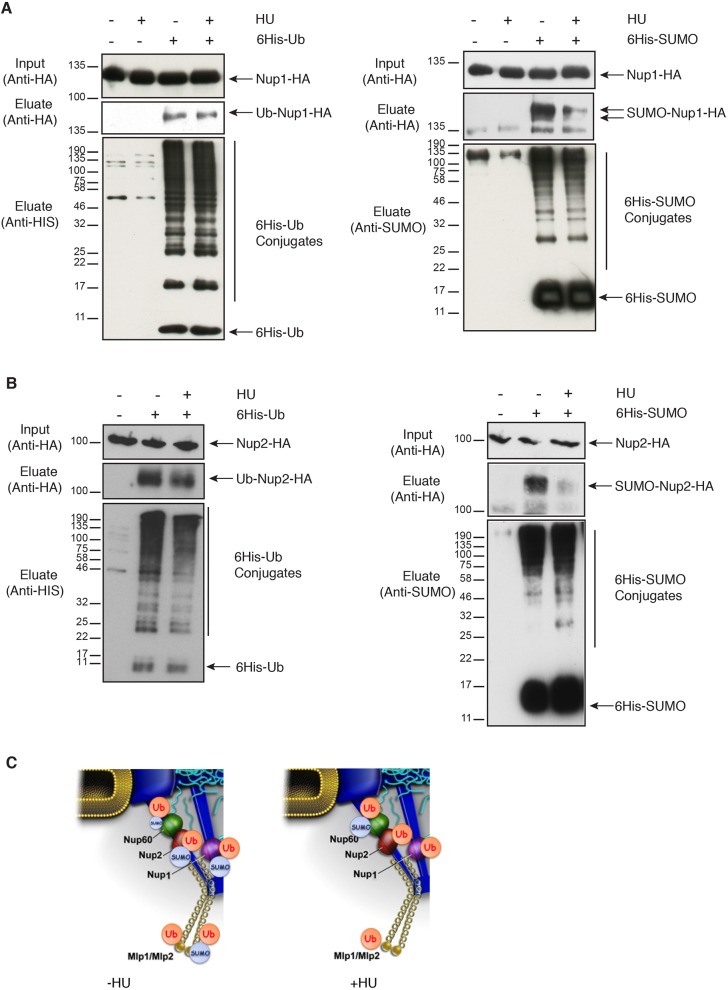
**Ubiquitylation and SUMOylation of Nup1 and Nup2.** (A,B) Ni-purified 6His–ubiquitin (Ub) or 6His–SUMO-conjugated forms of Nup1–HA (A) or Nup2–HA (B) were extracted from wild-type cells transformed (+) or not transformed (−) with a plasmid encoding 6His–ubiquitin (left panel) or 6His–SUMO (right panel) under control of the *CUP1* promoter, treated or not with 200 mM HU for 2 h. Cell lysates (input, top) and Ni-purified material (middle) were examined using western blotting with an anti-HA antibody. Ubiquitin and SUMO expression and efficiency of purification were controlled using an anti-His or anti-SUMO antibody, respectively (bottom) (*n*=3–5). (C) Recapitulative scheme of SUMO- and ubiquitin-conjugated species of nuclear basket nucleoporins without or with HU treatment.

For this reason, we carefully analyzed not only the SUMOylation but also the ubiquitylation patterns of genomically HA-tagged nuclear basket proteins, namely Nup1, Nup2 (Fig. 2A,B) and Mlps (Fig. S1) using the same experimental approaches as previously described (Fig. 1) (Niño et al., 2016). Similar to Nup60, all other nuclear basket nucleoporins, Nup1, Nup2, Mlp1 and Mlp2, were found conjugated to ubiquitin with a unique band corresponding to the modified protein, suggesting a monoubiquitylation event. However, treatment with HU did not alter the level of modification (Fig. 2A,B; Fig. S1).

Interestingly, SUMOylated species of Nup1, Nup2 and Mlp2 were also purified, with a drastic decrease observed upon HU treatment (Fig. 2A,B; Fig. S1). In contrast, Mlp1 was not SUMOylated even in a thermosensitive mutant of the NPC-associated SUMO protease Ulp1 (Fig. S1C). Thus, according to these observations, none of the nuclear basket Nups, beside Nup60, behave as typical DNA repair factors.

Taken together, these results illustrate that the post-translational modifications of nuclear basket proteins are extended rather than restricted to a specific nucleoporin and importantly, highly sensitive to stresses such as HU treatment (Fig. 2C).

### Dynamics of Nup60 and Nup2 SUMOylation

**Fig. 3.**
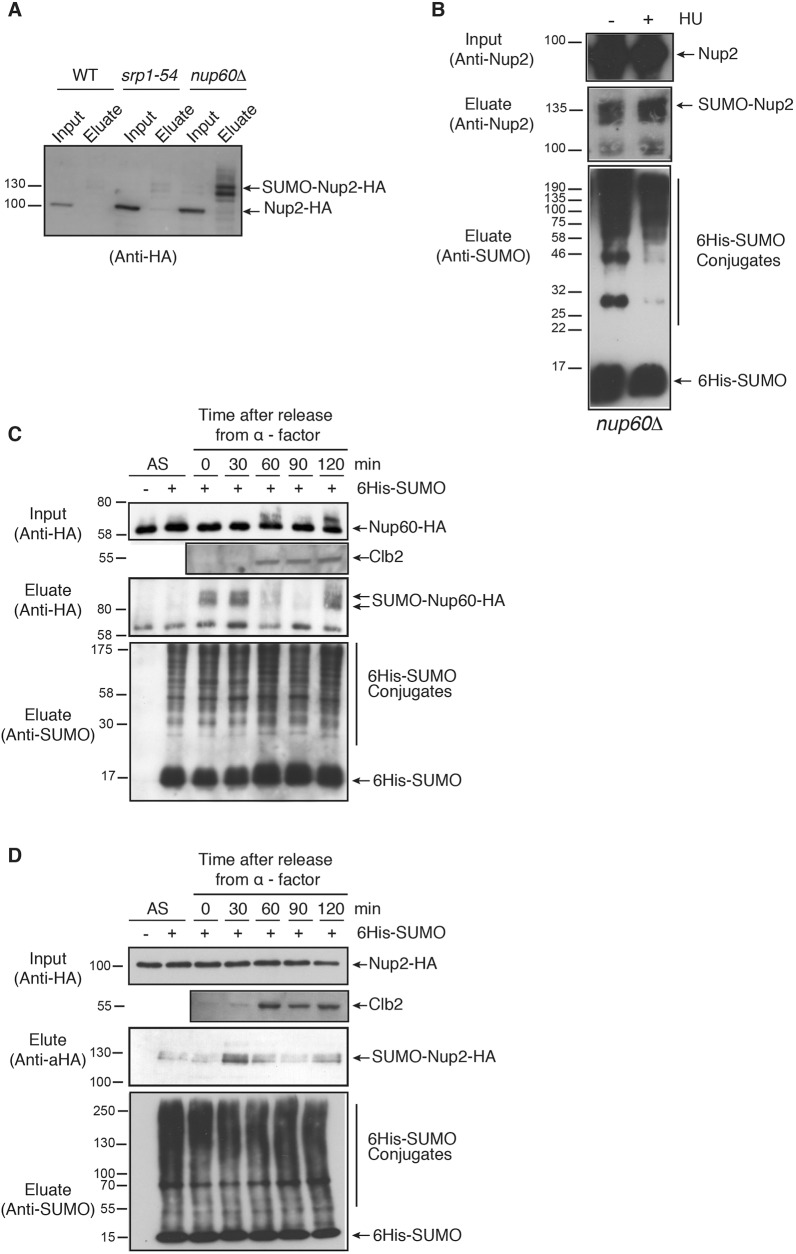
**Post-translational modifications of Nup60 and Nup2 during the cell cycle.** (A) Ni-purified 6His–SUMO-conjugated forms of Nup2 were extracted from wild-type (WT), or *srp1-54* or *nup60*Δ mutant cells transformed with a plasmid encoding 6His–SUMO under control of the *CUP1* promoter, and plasmid *UBC9*, encoding a SUMO conjugation enzyme to increase the efficiency of *in vivo* SUMOylation when SUMO is overexpressed. Cell lysates (input) and Ni-purified material (eluate) were examined using western blotting with an anti-HA antibody. Please note that a weak exposure is shown to indicate the increased SUMOylation in *nup60*Δ cells. As a consequence, Nup2 SUMOylation is not visible in wild-type cells (*n*=2). (B) Ni-purified 6His–SUMO-conjugated forms of Nup2 were extracted from *nup60*Δ cells transformed with a plasmid encoding 6His–SUMO under control of the *CUP1* promoter, with (+) or without (−) 200 mM HU treatment for 2 h and analyzed using western blotting with an anti-Nup2 antibody. SUMO expression and efficiency of purification was controlled using an anti-SUMO antibody. (C,D) Ni-purified 6His–SUMO-conjugated forms of Nup60–HA (C) and 6His–SUMO- or 6His-Ub-conjugated forms of Nup2–HA (D) were extracted from asynchronous cells (AS) or from cells treated with α-factor for 3 h before release for indicated periods of time. Cell lysates (top) and Ni-purified material (middle) were examined using western blotting with an anti-HA antibody, SUMO expression and efficiency of purification was controlled using an anti-SUMO antibody (bottom). The cell cycle progression was analyzed using an anti-Clb2 cyclin antibody in the cell lysates.

It was previously shown that Nup60 is responsible for the tethering of Nup2 at the NPC. Accordingly, Nup2 is localized in the nucleoplasm in the absence of Nup60 (Denning et al., 2001; Dilworth et al., 2001). *NUP60* deletion also leads to delocalization and destabilization of Ulp1 (Palancade et al., 2007). As shown in Fig. 3A, SUMOylation of Nup2 strongly increases in *nup60*Δ cells compared to *srp1-54* mutant (importin α mutant) cells with distinct SUMOylated species. This suggests that Nup2 is either more efficiently SUMOylated when not associated with the NPC, or alternatively more efficiently deSUMOylated at the NPC in a manner that is probably Ulp1-dependent, or both. Interestingly, SUMOylation of Nup2 remained stable upon HU treatment of *nup60*Δ cells (Fig. 3B), indicating that SUMOylation of nucleoplasmic Nup2 is not sensitive to HU treatment and favoring the hypothesis that Nup2 is strongly deSUMOylated at the NPC, particularly upon HU treatment.

HU-induced genotoxic stress results from a replicative stress that blocks cells in early S phase. The drastic consequences of HU treatment on the SUMOylation landscape of the nuclear basket nucleoporins (Figs 1 and 2) prompted us to analyze whether Nup60 and Nup2 post-translational modifications were affected during the cell cycle. In this respect, we recently reported that Nup60 was monoubiquitylated all through the cell cycle and highly phosphorylated in G2 by Rad53 kinase (Niño et al., 2016). Nup60 SUMOylation, which is barely detectable in non-synchronized cells unless the SUMO protease Ulp1 is deficient (Niño et al., 2016), was strongly enhanced upon α-factor-induced synchronization in G1, remained stable in S phase and no signal was detected from mitotic cells (Fig. 3C). A similar result was found in *rad53K227A* mutant cells (not shown). In addition, Nup60 SUMOylation was similarly induced upon HU-treatment in wild-type and *rad53K227A* mutant cells (Fig. 1B). Taken together, these data favor an induction of Nup60 SUMOylation upon genotoxic stress, independently of S-phase arrest.

In contrast to Nup60, the SUMOylation of Nup2 was detectable in non-synchronized cells and to the same extent upon synchronization in G1. However, SUMOylation strongly increased in S phase and then dropped in G2/M, as in Nup60 (Fig. 3D). The decrease of Nup2 SUMOylation upon HU treatment, compared to untreated and unsynchronized cells (Fig. 2B), thus probably results from the early S-phase arrest rather than a specific response to genotoxic stress.

Taken together, these data suggest that the balance between SUMOylation and deSUMOylation of Nup60 and of Nup2 differs greatly in the G1 and S phases.

### Mechanisms of Nup2 SUMOylation

**Fig. 4.**
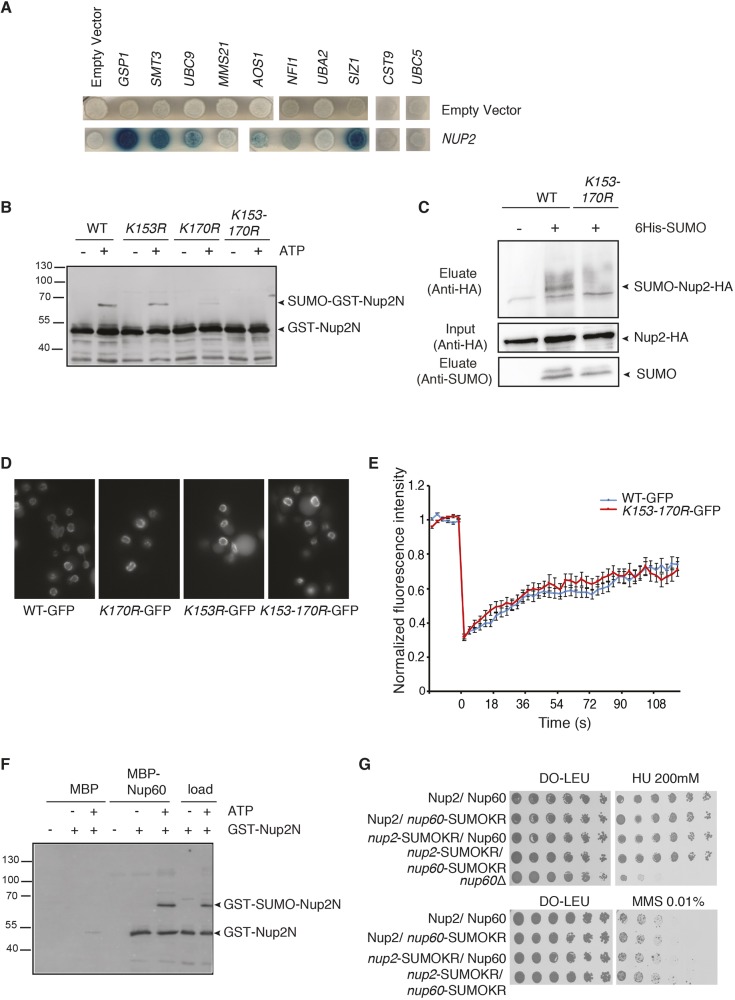
**SUMOylation of Nup2 at K153 and K170 does not regulate its interaction with the NPC.** (A) Two-hybrid assay was performed with a plasmid encoding LexA–Nup2 or with the LexA empty vector as a control and with B42 transactivator plasmids coding for various SUMO pathway fusion proteins as indicated. Expression of corresponding hybrids is shown in Fig. S2A. (B) *In vitro* SUMOylation of recombinant GST-conjugated Nup2 N-terminal residue 1–174 (GST–Nup2N) construct in its wild-type or mutated forms (K153R, K170R or combination of both) in the presence of E1, Ubc9 and Smt3, with (+) or without (−) treatment with 5 mM ATP as indicated. After SUMOylation, GST fusion proteins were purified using pulldown assays using glutathione sepharose. Bound proteins were analyzed using SDS-PAGE and western blotting using an anti-GST antibody. (C) *In vivo* SUMOylation of plasmid-encoded HA-tagged wild-type Nup2 or Nup2-*K153-170R*. Ni-purified 6His–SUMO-conjugated forms were extracted from cells transformed with plasmids encoding 6His–SUMO under control of the *CUP1* promoter, Ubc9, Nup2–HA or Nup2-*K153-170R*–HA. Cell lysates and Ni-purified material (eluate) were examined using western blotting with anti-HA or anti-SUMO antibodies. (D) Steady-state localization of GFP-tagged Nup2, Nup2-*K170R*, Nup2-*K153R* and Nup2-*K153-170R* in *nup2*Δ cells. (E) Average fluorescence recovery curves after photobleaching for WT–GFP (blue, *n*=38) and Nup2-*K153-170R*–GFP (red, *n*=47) cells. (F) *In vitro* pulldown assays using amylose agarose resin-coupled MBP or MBP–Nup60 and GST–Nup2N processed for SUMOylation *in vitro* with E1, Ubc9 and Smt3, with or without ATP. Bound proteins were analyzed using SDS-PAGE and western blotting using an anti-GST antibody. (G) Serial dilutions of wild-type and indicated mutant cell combinations were spotted on YPD plates without or with HU or MMS at the indicated concentrations and grown at 30°C.

To further understand the functions and regulation of Nup2 SUMOylation, mechanisms responsible for this post-translational modification were analyzed. A two-hybrid assay based on LexA- and B42-fused proteins revealed that besides its known partner Gsp1 (Ran GTPase), Nup2 interacts with SUMO (Smt3), the SUMO E1­-activating enzyme Aos1, the SUMO-conjugating enzyme Ubc9 as well as with the SUMO ligases Siz1 and Siz2. In contrast, no significant interaction was detected with the ubiquitin E2 Ubc5 and with the other SUMO E3 ligases Mms21 or Cst9, indicating that this nucleoporin is probably modified by the concerted action of SUMO E1, E2, Siz1 and Siz2 (Fig. 4A; Fig. S2A).

To map the lysine residues targeted for SUMO conjugation, various recombinant fragments of Nup2 fused to GST were assayed for *in vitro* SUMOylation in the presence of E1, E2 and SUMO (Smt3). A Nup2 region encompassing amino acid residues 85 to 174 was specifically shifted up in an ATP-containing reaction indicating a specific conjugation to SUMO (Fig. S2B). SUMO is conjugated to lysine residues usually in the context of a consensus site, ΨKXE/D, where Ψ is a large hydrophobic amino acid and X is any amino acid (Rodriguez et al., 2001). Two such motifs are indeed present in the Nup2^85–174^ fragment, centered on lysine residues K153 and K170. *In vitro* conjugation assays indicated that mutation of K153 or K170 into arginine led to a significant decrease of SUMOylation, whereas mutation of both completely abolished the modification of the corresponding recombinant GST-fusion proteins (Fig. 4B). In agreement, double mutation of these lysine residues also precluded *in vivo* SUMOylation of Nup2 (Fig. 4C) but not *in vivo* ubiquitylation (Fig. S3). Preventing SUMOylation of Nup2 neither altered its steady state localization at the NPC nor its dynamics at the NPC as assessed by mean of fluorescence recovery after photobleaching (FRAP) analysis (Fig. 4D,E). Finally, both SUMO-conjugated and -unconjugated Nup2 could interact *in vitro*, to the same extent, with recombinant Nup60 and recombinant importin α (Srp1) (Fig. 4F; Fig. S2C). In agreement with this, nuclear import of NLS-containing proteins, as well as the intracellular distribution of importin α, were not affected by Nup2 SUMOylation (Fig. S2D). Interestingly, both importin α (Solsbacher et al., 2000) and Nup60 (H.F., G.S., unpublished results) bind to the N-terminal domain of Nup2 (residues 1–174) and this domain also contains the SUMOylation sites.

To test whether Nup2 SUMOylation could synergize with Nup60 SUMOylation in the DNA damage response, mutations preventing Nup2 and/or Nup60 SUMOylation were combined. However, inhibiting SUMOylation of Nup60, Nup2 or a combination of both was not sufficient to alter cell sensitivity to HU or MMS (Fig. 4G).

Taken together, these data show that Nup2 is SUMOylated by Siz1 and Siz2 SUMO ligases on two distinct sites and probably de-SUMOylated mostly by Ulp1 at the NPC, but that this modification is involved in neither NPC tethering nor the NLS protein import pathway.

### SUMOylation of Nup2 at the NPC acts in the cellular response to osmostress

**Fig. 5.**
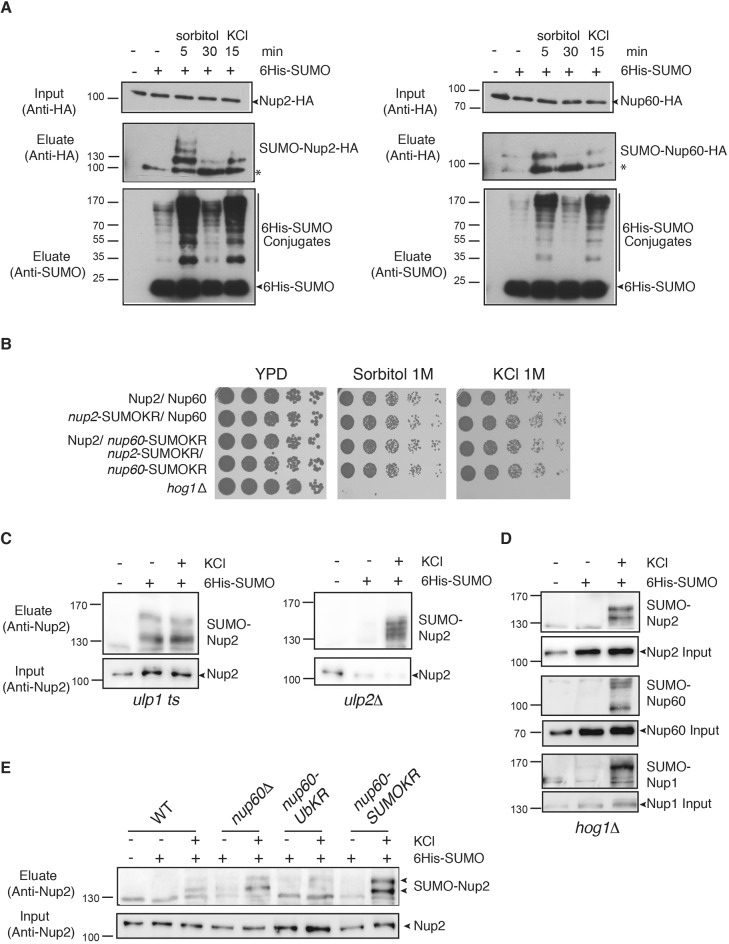
**SUMOylation of Nup2 and Nup60 is sensitive to osmostress.** (A) Ni-purified 6His–SUMO-conjugated forms of Nup2–HA (left panel) or Nup60–HA (right panel) were extracted from cells transformed with a plasmid encoding 6His–SUMO under control of the *CUP1* promoter and treated (+) or not (−) with 1 M sorbitol (for 5 or 30 min) or 1 M KCl for 15 min and analyzed as in Fig. 1. * indicates deconjugated Nup2–HA. (B) Serial dilutions of wild-type and indicated mutant cells were spotted on YPD plates without or with 1 M sorbitol or 1 M KCl and grown at 30°C. (C–E) Ni-purified 6His–SUMO-conjugated forms of untagged Nup2 (and when indicated Nup60 and Nup1) were extracted from (C) *ulp1 ts* or *ulp2*Δ mutant cells, (D) *hog1*Δ cells as well as (E) wild-type or *nup60* mutant cells carrying a *UBC9* plasmid, treated or not with 1 M KCl for 15 min, and analyzed with an anti-Nup2 antibody (C–E) or with anti-Nup60 and anti-Nup1 (D) (*n*=3).

It has been reported that three nucleoporins from yeast, Nup1, Nup2 and Nup60, are phosphorylated by the Hog1 osmostress activated-protein kinase (Regot et al., 2013). To analyze whether osmostress affects not only phosphorylation but could also induce SUMOylation of these Nups, modification of Nup60 and Nup2 was analyzed upon 15 min KCl (1 M) or 5 min and 30 min sorbitol (1 M) treatments. These treatments dramatically increased SUMOylation of both Nup2 and Nup60, with distinct SUMOylated bands clearly identified for Nup2 (Fig. 5A). It should be noted that, more generally, the osmotic stresses led to a strong increase of overall SUMOylation of cellular proteins (see Fig. 5A, lower panels) as previously shown (Abu Irqeba et al., 2014; Oeser et al., 2016; Lewicki et al., 2015). In addition, SUMO response to sorbitol was transient as a 30-min treatment was no more effective in terms of SUMOylation induction than a 5-min treatment, as expected for a specific osmostress response. However, deletion of *NUP2* or *NUP60* does not alter cell sensitivity to osmostress-inducing conditions such as 1 M NaCl or 2 M sorbitol (Regot et al., 2013; C.A.N., S.T., H.F., G.S., C.D., unpublished observations) and combination of SUMO sites mutations in Nup2 and/or Nup60 did not affect osmostress sensitivity (Fig. 5B). This suggests that SUMOylation of these Nups is a strong stress sensor but not essential for the proper stress response.

This osmostress sensitivity was abrogated in a *ulp1 ts* mutant whereas it was not altered upon *ULP2* deletion (Fig. 5C). Interestingly, increased SUMOylation of Nup2 in *nup60*Δ cells could still be enhanced upon osmostress (Fig. 5E; Fig. S4). Deletion of *NUP60* not only led to Nup2 delocalization to the cytoplasm but also to destabilization and delocalization of Ulp1. This indicates that osmostress induced increased SUMOylation at the NPC rather than preventing deSUMOylation. Interestingly, the KCl-induced SUMOylation of not only Nup2, but also Nup60 and Nup1, still occurred in the absence of Hog1, and was persistent instead of transient (Fig. 5D and not shown), thus demonstrating that phosphorylation and SUMOylation of Nup2 are targets of both canonical and non-canonical osmostress pathways, respectively (Hohmann, 2015). Finally, preventing SUMOylation of Nup60 strongly synergized with the osmostress-induced Nup2 SUMOylation (compared to wild-type cells or ubiquitylation-deficient Nup60 mutant cells), thus further exemplifying the crosstalk between both nucleoporins (Fig. 5E; Fig. S4).

## DISCUSSION

The eukaryotic cell is host to a multitude of metabolic activities at any point in time, which calls for a sophisticated system of coordination to maintain cell viability and health. A core component of this coordination is the presence of cellular hubs where metabolic processes converge to create a platform for intracellular crosstalk. The NPC is one such platform that coordinates nucleocytoplasmic transport, gene regulation and DNA repair processes. However, how the NPC coordinates these metabolic activities is not well understood. We have previously reported that post-translational modifications of NPC proteins are important for its function in various nuclear processes. In particular, we found that ubiquitylation of the nuclear basket protein Nup60 is enhanced by genotoxic stress and stabilizes the interaction of Nup60 and its partner Nup2 with Nup84 – a component of the Y complex – and consequently with the NPC. Ubiquitylated Nup60 is a target for Rad53 kinase and plays a role in the DNA damage response initiated by replication stress. In the present study, we evaluated whether SUMOylation of Nup60 and Nup2 is responsive to genotoxic stress and, more generally, to other cellular stress signals.

First, we evaluated whether the SUMOylation of Nup60 is affected by genotoxic stress. We found that exposure to replication stress by treatment with HU or MMS induced Nup60 SUMOylation independently of the canonical DNA damage pathways. Nup60 is thus revealed to be a target of the genotoxic stress response, acting in both the Rad53-mediated (Niño et al., 2016) and possibly in the SUMO-mediated response as a function of its modification by ubiquitin and SUMO, respectively. The involvement of the NPC nuclear basket in the maintenance of genomic integrity is probably conserved in eukaryotes, as the Nup60 ortholog Nup153 is not only SUMOylated but also essential for the proper activation of the DNA damage checkpoint in human cells (Chow et al., 2012; Lemaître et al., 2012). However, this is not a common property of nuclear basket proteins as, in contrast to Nup60, we observed a significant reduction in SUMOylation levels of Nup1, Nup2 and Mlp2 upon HU treatment. In addition, preventing SUMOylation of both Nup60 and Nup2 did not sensitize cells to HU or MMS treatment indicating that, in contrast to *stricto sensu* repair factors, simultaneous mutations of SUMO sites on nuclear basket nucleoporins is not sufficient to induce a major DNA damage response defect (Psakhye and Jentsch, 2012).

Although Nup60 and Nup2 are both direct interaction partners and SUMOylated, we observed that the regulation of their modification varies not only during genotoxic stress but also during the cell cycle. In synchronized cells, Nup60 SUMOylation indeed increased in G1 and remained stable in S phase of the cell cycle, while Nup2 showed enriched SUMOylation in the S phase. This regulation is probably the result of a differential balance between Siz1- and/or Siz2-dependent SUMOylation and Ulp1-dependent deSUMOylation at the NPC. Whether such rearrangements of SUMO marks ensure a continual tuning of SUMO level at the nuclear basket and provide a sensing mechanism similar to the role of SUMO in sensing and signaling DNA lesions would be an interesting model to explore (Garvin and Morris, 2017). Interestingly, both Nup60 and Nup2 displayed a drop in SUMOylation in the G2/M phase that could, at least partially, be explained by the nuclear export of the major SUMO E3 ligase Siz1 and nuclear degradation of remaining nuclear Siz1 (Makhnevych et al., 2007; Westerbeck et al., 2014).

Given that Nup1, Nup2 and Nup60 are phosphorylated by the Hog1 protein kinase upon osmostress (Regot et al., 2013), we analyzed SUMOylation of Nup2 and Nup60 upon osmostress and observed a dramatic increase in their SUMOylation levels and even polySUMOylation of Nup2. Hyperosmotic stresses have been reported to cause rapid and transient Siz1-dependent SUMOylation of yeast cellular proteins (Abu Irqeba et al., 2014; Oeser et al., 2016; Lewicki et al., 2015). However, the modification of NPC proteins was stress-specific as ethanol stress, which also results in increased SUMOylation, had no effect on nuclear basket Nup2 (not shown). As neither *NUP2* nor *NUP60* deletion renders the cell sensitive to osmostress, the role of Nup2 and Nup60 SUMOylation in this stress adaptation is unclear. However, we found that Nup2 and Nup60 SUMOylation in response to KCl treatment proceeded even in the absence of Hog1 kinase. Besides its ability to regulate gene expression via the phosphorylation of specific transcription factors such as Hot1 (Saito and Posas, 2012), Hog1 has indeed been proposed to promote osmotolerance by limiting the accumulation of abnormal SUMOylated species (Abu Irqeba et al., 2014). This suggests that phosphorylation and SUMOylation of Nup2 and Nup60 engage in the canonical and non-canonical pathways in osmostress. Interestingly, we observed an enrichment of Nup2 SUMOylation upon osmostress in the unSUMOylatable *nup60* mutant. Would it participate in the continual tuning of SUMO levels at the nuclear basket as proposed above? In conclusion, data presented here indicate that the crosstalk between nuclear basket nucleoporins is multilayered, and speaks to our hypothesis that NPC acts as a sensor of various stresses.

## MATERIAL AND METHODS

### Yeast strains, plasmids, and cell culture

The *Saccharomyces cerevisiae* strains and plasmids used in this study are listed in Tables S1 and S2. Yeast cultures were grown at 30°C either in YPD media containing 2% glucose or in synthetic media (SD) with appropriate supplements. Cell growth assays were performed by fivefold serial dilutions of the different strains spotted on YPD plates without or with HU or MMS at the indicated concentrations and grown at the indicated temperatures. For drug sensitivity analysis, cells were incubated for 90 min at 30°C in the presence of MMS (0.02%) or HU (200 mM).

The derivative strains (chromosomally tagging and deletion mutants) were constructed using PCR-based homologous recombination (Longtine et al., 1998). The genomic integration of the *nup2-*SUMO-KR mutations and the *NUP2* wild-type control was achieved by transformation of the *nup2*Δ strain with linearized integration plasmid. The integration at the correct locus was verified using control PCRs and western blot analysis. The KR point mutations were generated with the QuickChange site-directed mutagenesis kit (Stratagene).

### Purification of ubiquitylated and SUMOylated proteins, *in vitro* SUMOylation assays and protein purification

Cells transformed with a plasmid encoding 6His–ubiquitin or 6His–SUMO under the *CUP1* promoter were grown on selective medium and stimulated overnight with 0.1 mM CuSO_4_. Purification of ubiquitylated and SUMOylated proteins was performed on Ni-NTA agarose beads (Qiagen) as previously described (Hayakawa et al., 2012) and improved in Niño et al. (2016). Proteins were analyzed using western blotting using anti-HA (Biolegend, HA-11, 0.75 μg ml^−1^), anti-His tag (Millipore, 70796, 0.2 μg ml^−1^), polyclonal rabbit anti-Smt3 (a gift of Benoit Palancade, Institut Jacques Monod, Paris, France; 1:10,000), polyclonal rabbit anti-Clb2 (gift from Carl Mann, I2BC, Saclay, France; 1:500) or affinity-purified rabbit anti-Nup2 antibodies generated in-house (made against Nup2 N-terminus, residues 1–174), anti-full-length Nup60, or anti-Nup1 N-terminus (residues 24–287) all at 1:500 for western blotting. When indicated, cells were also transformed with pRS423-*UBC9* or pRS424-*UBC9* to increase the SUMOylation efficiency. Protein purifications, pulldown assays, and *in vitro* SUMOylation assays were performed as previously described (Rothenbusch et al., 2012). The results shown in the figures are representative of three to five independent experiments as indicated in each figure legend.

### Two-hybrid assays

Two-hybrid assays were performed using the DupLEX-A system (OriGene Technologies, Rockville, MD) as previously described (Caesar et al., 2006; Rothenbusch et al., 2012). Briefly, EGY48 cells containing a LexA operon–*LEU2* reporter transformed with the empty vector pEG202 or the bait plasmid pEG–Bam–*NUP2* encoding the LexA DNA-binding domain fused to Nup2 were mated with strains containing various pJG4–5-derived prey vectors containing *GAL1* promoter-driven B42 activation domain fusions to SUMOylation factors. The cells were spotted onto agar plates with synthetic complete histidine- and leucine-deficient media and incubated at 30°C for 2 d. The synthesis of the hybrid proteins was confirmed by use of western blotting with anti-LexA (Santa Cruz Biotechnology, 2-12; 1:500) and anti-HA (Biolegend, HA-11, 0.75 μg ml^−1^) antibodies (Fig. S2).

### Cell synchronization and analysis

*bar1*Δ cells grown at either 25°C or 30°C were synchronized in G1 by means of treatment with α-factor (30 nM final concentration) for 3 h, and cells were collected at different time points after release in fresh medium. Flow cytometry analysis was performed as previously described (Hayakawa et al., 2012).

### Fluorescence microscopy

Yeast cells were viewed using direct fluorescence microscopy with an Observer. Z1 microscope (magnification 1000×, Carl Zeiss) and processed with the program Axio Vision 4.8.2 SP1.

### Fluorescence recovery after photobleaching

FRAP experiments were performed and analyzed as previously described (Niño et al., 2012) with a spinning-disk confocal on a fully motorized inverted microscope (Eclipse Ti-E; Nikon) controlled with MetaMorph software 7.7.8, equipped with the Perfect Focus System (Nikon), a 100×, 1.45 NA Plan Apochromat oil immersion objective, a piezo stage (Mad City Labs), a spinning-disk unit (CSUX1; Yokogawa), a charge-coupled device camera (CoolSNAP HQ2, Photometrics-Princeton), and a laser bench (Roper Scientific, France) with 491-nm and 561-nm diode lasers (100 mW each; Cobolt). Images were registered and analyzed with ImageJ (plugin TurboReg and Curve Fitting Tool).

## Supplementary Material

Supplementary information
