# Supplementary material for: SUMOylation of the nuclear pore complex basket is involved in sensing cellular stresses
Source: J Cell Sci. 2019 Apr 3;132(7):jcs224279. doi: 10.1242/jcs.224279 (PMC6467484; doi:10.1242/jcs.224279)
Supplement: Supplementary information [file joces-132-224279-s1.pdf]

Table S1. *S. cerevisiae* strains used in this study

Strain	Genotype	Reference
EGY48	MATα *ura3 leu2 his3 trp1 lexA op::LEU2*	OriGene
W303	MAT*a ura3-1 leu2-3, 112 trp1-1 ade2-1 his3-11,15 can1-100*	wild-type
Δnup2	MATα *leu2-3,112 trp1-1 ade2-1 NUP2::ura3-1::HIS3* (GSY432)	(Loeb et al., 1993)
srp1-54	MAT*a ura3 leu2 his3 trp1 ade2 can1* (GSY664)	(Hahn et al., 2008)
Δulp2	MAT*a trp1Δ63 his3Δ200 leu2-3,112 lys2-810 ura3-52 ulp2-1* (IS18-M2)	(Schwienhorst et al., 2000)
ulp1-333 ts	MAT*a ulp1::HIS3 LEU2::ulp1-333 ura3-52 lys2-801 trp1-1 his3Δ200*	(Li et al., 1999)
Δnup60	*NUP60::TRP1* MAT*a ura3-1 leu2-3, 112 ade2-1 his3-11,15*	This study
NUP2	MATα *leu2-3,112 trp1-1 ade2-1 Δnup2::HIS3 NUP2::URA3*	This study
NUP2-SUMO-KR	MATα *leu2-3,112, trp1-1, ade2-1, Δnup2::HIS3 NUP2-K153R-K170R::URA3*	This study
BY4741	*MATa his3Δ1 leu2Δ0 met15Δ0 ura3Δ0*	Euroscarf wild-type
Δhog1	*MATa his3Δ1 leu2Δ0 met15Δ0 ura3Δ0 HOG1::KANMX6*	Gift from V. Panse
nup60-Ub-KR	MAT*a his3Δ1 leu2Δ0 met15Δ0 ura3Δ0 nup60-K105R-K175R-HA::LEU2*	(Nino et al., 2016)
nup60-SUMO-KR	MAT*a his3Δ1 leu2Δ0 met15Δ0 ura3Δ0 nup60-K(440-442-505)R-HA::LEU2*	(Nino et al., 2016)
nup60-HA	*MATa his3Δ1 leu2Δ0 met15Δ0 ura3Δ0 nup60-HA::HIS3*	(Nino et al., 2016)
nup1-HA	*MATa his3Δ1 leu2Δ0 met15Δ0 ura3Δ0 nup1-HA::HIS3*	This study
nup2-HA	*MATa his3Δ1 leu2Δ0 met15Δ0 ura3Δ0 nup2-HA::HIS3*	This study
mlp1-HA	*MATa his3Δ1 leu2Δ0 met15Δ0 ura3Δ0 mlp1-HA::HIS3*	This study
mlp2-HA	*MATa his3Δ1 leu2Δ0 met15Δ0 ura3Δ0 mlp2-HA::HIS3*	This study

nup60-HA Δbar1	*MATa his3Δ1 leu2Δ0 met15Δ0 ura3Δ0 nup60-HA::LEU2 BAR1::HPH*	(Nino et al., 2016)
nup2-HA Δbar1	*MATa his3Δ1 leu2Δ0 met15Δ0 ura3Δ0 nup2-HA::HIS3 BAR1::HPH*	This study
NUP2-SUMO-KR Δnup60	MATα *leu2-3,112, trp1-1, ade2-1, Δnup2::HIS3, NUP2-K153R-K170R::URA3, Δnup60::HPH*	This study
nup60-HA Δmre11	*nup60-HA::LEU2 mre11:: KANMX6*	This study
rad53K227A bar1Δ nup60 HA	*MATa his3Δ1 leu2Δ0 met15Δ0 ura3Δ0, nup60-HA:LEU2, BAR1::HPH, rad53K227A::KANMX6*	(Nino et al., 2016)
rad53K227A bar1Δnup60-HA SUMO-KR	*MATa his3Δ1 leu2Δ0 met15Δ0 ura3Δ0, nup60-K(440,442,505)R -HA:LEU2, BAR1::HPH, rad53K227A::KANMX6*	This study

Table S2. Plasmids used in this study

**Plasmid**	**Encoded protein**	**Origin**
pRS314-*SRP1-GFP* (pGS287)	Srp1-GFP	This study
YCpGAL-*NLS-GST-GFP* (pGS422)	NLS-GST-GFP	(Solsbacher et al., 1998)
pRS316-*NUP2-GFP* (pGS583)	Nup2-GFP	This study
pGEX-4T-*NUP2-N1* (pGS743)	GST-Nup2$^{2-84}$	This study
pGEX-4T-*NUP2-N2* (pGS744)	GST-Nup2$^{85-174}$	This study
pGEX-4T-*NUP2-N3* (pGS1169)	GST-Nup2$^{43-127}$	This study
pGEX-4T-*NUP2-N* (pGS815)	GST-Nup2$^{1-173}$	(Solsbacher et al., 2000)
pGEX-4T-*NUP2-M* (pGS278)	GST-Nup2$^{175-563}$	(Solsbacher et al., 2000)
pGEX-4T-*NUP2-C* (pGS273)	GST-Nup2	(Solsbacher et al., 2000)
pJG-*GSP1* (pGS893)	B42 AD-Gsp1	(Caesar et al., 2006)
pJG-Bam-*UBC5* (pGS1292)	B42 AD-Ubc5	This study
pJG-*NFI1* (pGS 1781)	B42 AD-Nfi1	(Rothenbusch et al., 2012)
pJG-Bam-*SMT3* (pGS1892)	B42 AD-Smt3	(Rothenbusch et al., 2012)
pJG-Bam-*UBA2* (pGS1894)	B42 AD-Uba2	(Rothenbusch et al., 2012)
pJG-Bam-*AOS1* (pGS1895)	B42 AD-Aos1	(Rothenbusch et al., 2012)
pJG-Bam-*UBC9* (pGS1897)	B42 AD-Ubc9	(Rothenbusch et al., 2012)
pJG-Bam-*SIZ1* (pGS1900)	B42 AD Siz1	(Rothenbusch et al., 2012)
pJG-Bam-*MMS21* (pGS1902)	B42-AD-Mms21	(Rothenbusch et al., 2012)
pEG-*NUP2* (pGS1973)	lexA BD-Nup2	This study
pJG-Bam-*CST9* (pGS1984)	B42 AD-Cst9	(Rothenbusch et al., 2012)

pRS426-*NUP2-3xHA* (pGS2010)	Nup2-3xHA	This study
pGEX-4T-*NUP2-N K153R* (pGS2041)	GST-Nup2-N K153R	This study
pGEX-4T-*NUP2-N K170R* (pGS2042)	GST-Nup2-N K170R	This study
pRS316-*NUP2-GFP K170R* (pGS2084)	Nup2-GFP K170R	This study
pGEX-4T-*NUP2-N K153R K170R* (pGS2087)	GST-Nup2-N K153R K170R	This study
pRS426-*NUP2-HA K153R K170R* (pGS2117)	Nup2-HA K153R K170R	This study
pRS316-*NUP2-GFP K153R* (pGS2120)	Nup2-GFP K153R	This study
pRS316-*NUP2-GFP K153R K170R* (pGS2121)	Nup2-GFP K153R K170R	This study
pRS306-NUP60-HA-SUMOKR	Nup60-SUMOKR-HA	(Nino et al., 2016)
pRS423-*UBC9* (pGS2169)	Ubc9	This study
pRS424-*UBC9* (pGS2170)	Ubc9	This study
pMAL-C2-*NUP2-N* (pGS2210)	MBP-Nup2-N	This study
pMAL-C2-*NUP60* (pGS2235)	MBP-Nup60	This study
YEp351-*pCUP1-6His-SMT3* (pGS2311)	6His-SUMO	Gift from B. Palancade
YEp352-*pCUP1-6His-SMT3*	6His-SUMO	(Nino et al., 2016)
YEp351-*pCUP1-6His-Ub* (pGS2307)	6His-Ubiquitin	Gift from Jürgen Dohmen
YEp352-*pCUP1-6His-Ub*	6His-Ubiquitin	(Nino et al., 2016)

Abbreviations, AD activation domain, BD DNA binding domain

Journal of Cell Science • Supplementary information

Supplementary Figures

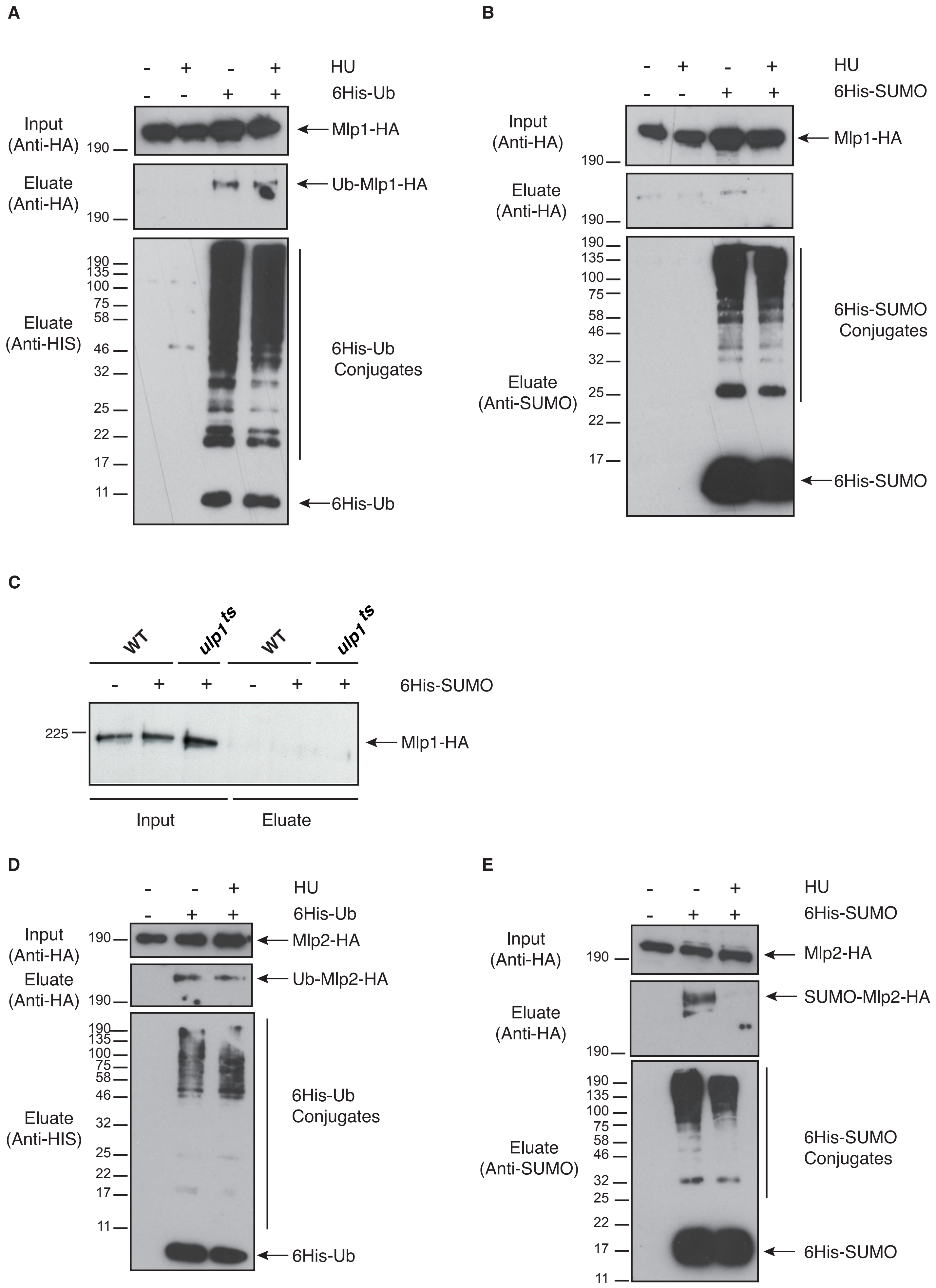

Journal of Cell Science • Supplementary information

Figure S1. Ubiquitylation and SUMOylation of Mlp1 and Mlp2. Ni-purified 6His-ubiquitin (Ub) or 6His-SUMO–conjugated forms of Mlp1-HA **(A, B, C)** or Mlp2-HA **(D, E)** were extracted from wild-type or *ulp1 ts* cells transformed (+) or not transformed (−) with a plasmid encoding 6His-ubiquitin (**A, D**) or 6His-SUMO (**B, C, E**) under control of the *CUP1* promoter, treated or not with 200 mM HU for 2 hours. Cell lysates (top) and Ni-purified material (middle) were examined by Western blotting with an anti-HA antibody. Ubiquitin and SUMO expression and efficiency of purification was controlled using an anti-6His or anti-SUMO antibody respectively (bottom) (n=2).

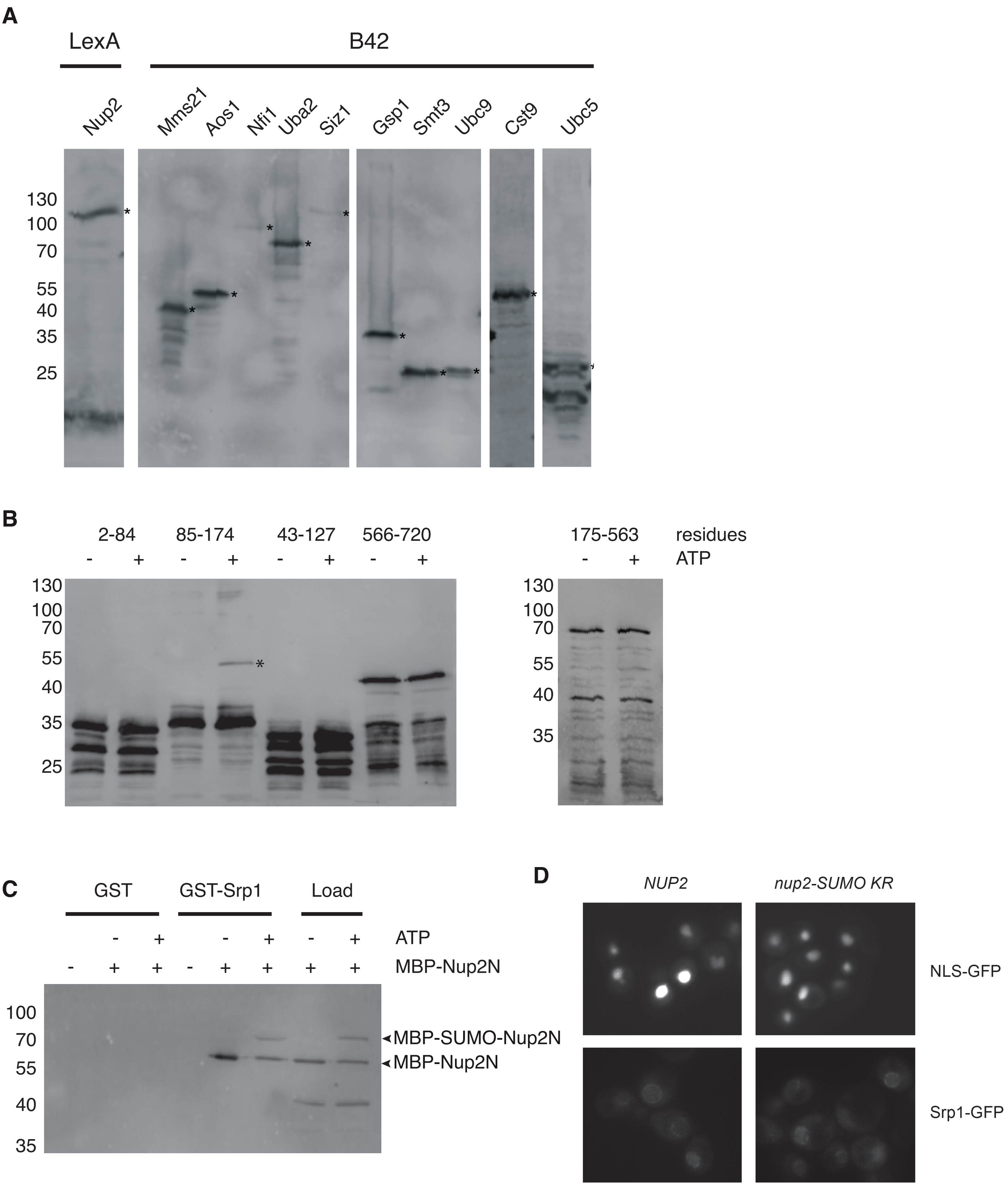

Journal of Cell Science • Supplementary information

Figure S2. (A) Expression of fusion proteins used in two-hybrid assays shown in Figure 4A was analyzed by Western blotting using affinity-purified LexA or HA-specific antibodies. * indicate fusion proteins of the expected size. **(B)** *In vitro* SUMOylation of recombinant GST fused to various Nup2 fragments in the presence of E1, Ubc9, Smt3 +/- ATP as indicated. After SUMOylation, GST fusion proteins were purified by pulldown assays using glutathione sepharose. Bound proteins were analyzed by SDS-PAGE and Western blotting using GST-specific antibodies. * indicates the SUMOylation product appearing for $Nup2^{85\text{-}174}$. **(C)** *In vitro* pulldown assays using glutathione-coupled GST or GST-Srp1 and MBP-Nup2N processed for SUMOylation with E2, Ubc9 and Smt3 +/-ATP. Bound proteins were analyzed by SDS-PAGE and Western-blotting using anti MBP antibodies. **(D)** Localization of the NLS-GST-GFP import reporter and of Srp1 (importin α) was analyzed in WT and Nup2-K153-170R cells. The strains were transformed with plasmids coding for Srp1-GFP or NLS-GST-GFP. The steady state localization was monitored for importin α. The GFP-tagged classical NLS import reporter was analyzed after galactose induction for 2 hours.

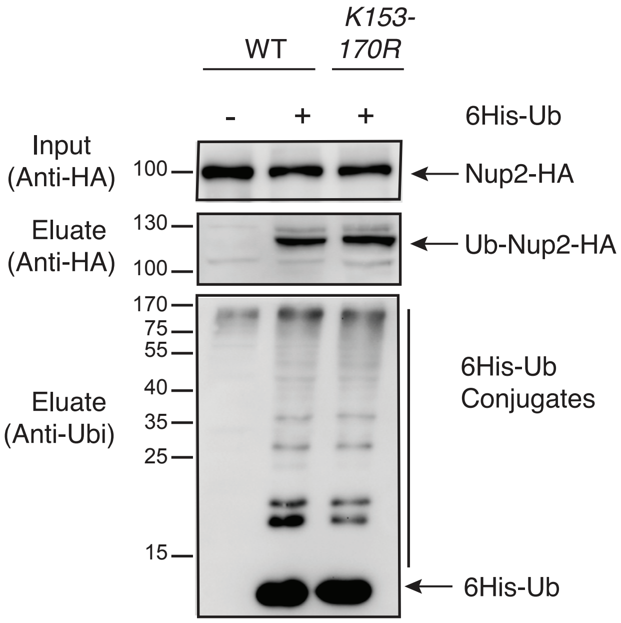

Figure S3. *In vivo* ubiquitylation of plasmid-encoded HA-tagged wt or K153-170R Nup2. Ni-purified 6His-Ub conjugated forms were extracted from cells transformed with plasmids encoding 6His-Ub under control of the *CUP1* promoter, Nup2-HA or Nup2-*K153-170R*-HA. Cell lysates and Ni-purified material (eluate) were examined by Western blotting with anti-HA or anti-His antibodies.

Journal of Cell Science • Supplementary information

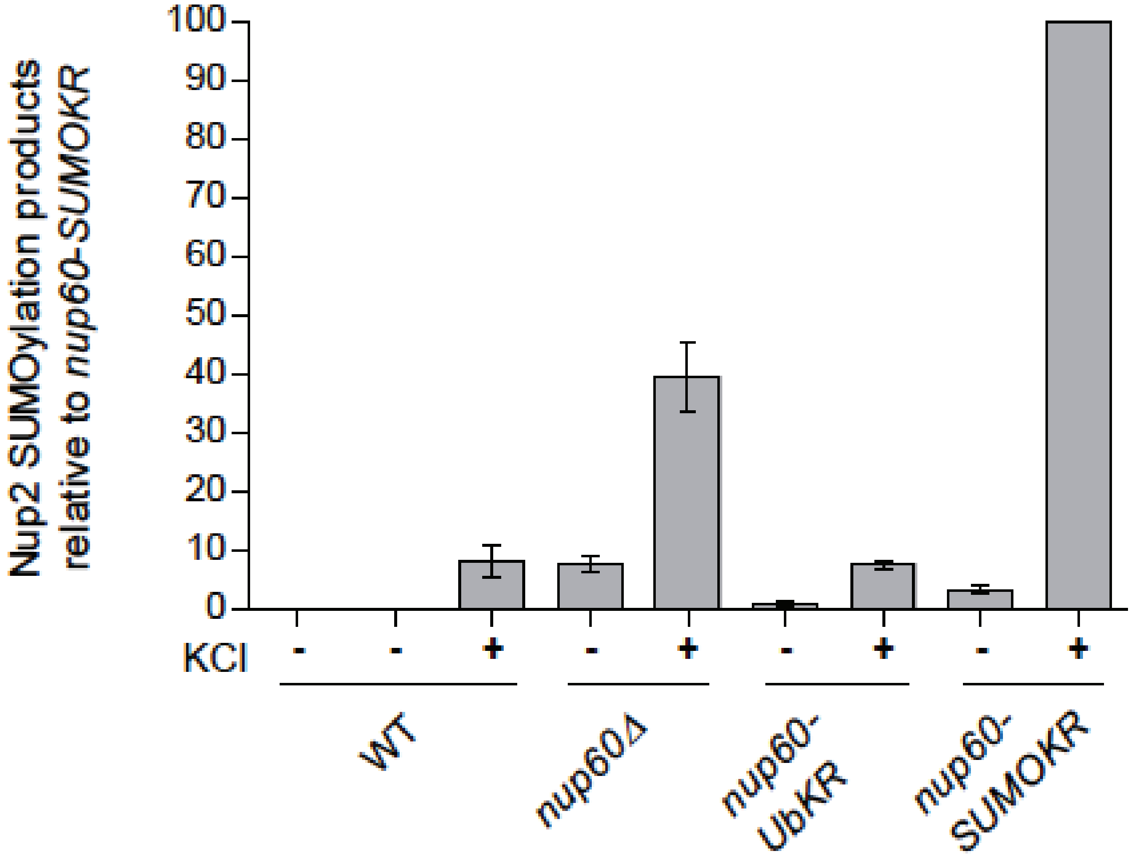

Figure S4. Quantification of Figure 5E. The amounts of Nup2-SUMO products in the different strains were quantified from 3-4 experiments and normalized to the inputs. The SUMO products in the different strains are represented as % of the value in the *nup60-SUMO KR* mutant, which was set to 100 % (mean+/-sem).

References

Caesar, S., Greiner,M. and Schlenstedt, G. (2006). Kap120 functions as a nuclear import receptor for ribosome assembly factor Rpf1 in yeast. *Mol. Cell. Biol.* **26,** 3170-3180. doi:10.1128/MCB.26.8.3170-3180.2006

Hahn, S., Maurer, P., Caesar, S. and Schlenstedt, G. (2008). Classical NLS proteins from Saccharomyces cerevisiae. *J. Mol. Biol.* **379**, 678-694. doi:10.1016/j.jmb.2008.04.038

Li, S. J. and Hochstrasser, M. (1999). A new protease required for cell-cycle progression in yeast. *Nature* **398**, 246-251. doi:10.1038/18457

Loeb, J. D., Davis, L. I. and Fink, G. R. (1993). NUP2, a novel yeast nucleoporin, has functional overlap with other proteins of the nuclear pore complex. *Mol. Biol. Cell* **4**, 209-222. doi:10.1091/mbc.4.2.209

Niño, C. A., Guet, D., Gay, A., Brutus, S., Jourquin, F., Mendiratta, S., Salamero, J., Geli, V. and Dargemont, C. (2016). Posttranslational marks control architectural and functional plasticity of the nuclear pore complex basket. *J. Cell Biol.* **212**, 167-180. doi:10.1083/jcb.201506130

Rothenbusch, U., Sawatzki, M., Chang, Y., Caesar, S. and Schlenstedt, G. (2012). Sumoylation regulates Kap114-mediated nuclear transport. *EMBO J.* **31**, 2461-2472. doi:10.1038/emboj.2012.102

Schwienhorst, I., Johnson, E. S. and Dohmen, R. J. (2000). SUMO conjugation and deconjugation. *Mol. Gen. Genet.* **263**, 771-786. doi:10.1007/s004380000254

Solsbacher, J., Maurer, P., Bischoff, F. R. and Schlenstedt, G. (1998). Cse1p is involved in export of yeast importin alpha from the nucleus. *Mol. Cell. Biol.* **18**, 6805-6815. doi:10.1128/MCB.18.11.6805

Solsbacher, J., Maurer, P., Vogel, F. and Schlenstedt, G. (2000). Nup2p, a yeast nucleoporin, functions in bidirectional transport of importin alpha. *Mol. Cell. Biol.* **20**, 8468-8479. doi.:10.1128/MCB.20.22.8468-8479.2000

Journal of Cell Science • Supplementary information
